# The Nurses' Tribute to the Dead Prince

**Published:** 1892-01-23

**Authors:** 


// The Hospital

A Weekly Journal of

Science, Medicine, Nursing, and Philanthropy.

*For Hospitals, Asylums, Infirmaries, Dispensaries, Medical Practitioners, Students, Nurses, and the Charitable Public.*

Vol. XI.—No. 278.] [Jan. 23, 1892.

### THE NURSES' TRIBUTE TO THE DEAD PRINCE.

In the sorest of straits, in the deepest of griefs there is help and consolation in the sympathy of one human heart for another. The instinct of the deeply wounded soul is to sit apart, and abandon itself to hopeless sorrow: and the instinct of sympathising love is to respect the sacredness of such a desire for solitude, and to refrain from offering consolation. Btut there are times when affection is constrained to break through the barriers of even the most sacred loneliness of grief. The present is such a time for many of those women who have given up their lives to the nursing of the sick. They love the Princess of Wales as an exalted friend and sister. If any one were to forbid such love, and to say that respect, the respect of the comparatively lowly for the exalted is more fitting, the nurses would have to say that the love they feel for the Princess of Wales has sprung up in their hearts spontaneously and of itself, and that whether it be or be not approved by a cold conventionalism, it is in their hearts, and they cannot, so long as the Princess is the Princess, tear it up by the roots.

The affection which so many nurses feel for her Royal Highness has had its beginnings in herself, and in herself alone. She has stooped from her high station, like some queen of a great poet's dream, to meet half-way and to encourage in their work those gentle womanly spirits who minister to the sick, the sorrowing, and the dying. If the Princess had a heart to be touched with the work done by nurses, before her experiences of the past few months, how much more does she understand to-day of the noble anxieties and tender ministrations of the chamber of suffering and death!

Very naturally the Nurses of the Royal National Pension Fund first, but after them all other good women in the nursing world, longed with a heartfelt longing to say some word or do some deed which should show the Princess of Wales and the Prince, and the Princess May, how deeply and even painfully their hearts were moved by the almost unparalleled calamity which has befallen the Royal House. Very naturally also they could not bear the thought that their sympathy if offered might be felt as a distressing intrusion. But for them to sit silent as if the Princess's grief were no concern of theirs was impossible. Large numbers of them wrote to the founder of the National Pension Fund, begging him in terms whose urgency could not be resisted to take some immediate steps—they did not know, and could hardly suggest what—in order that Her Royal Highness the Princess, and the Royal household at Sandringham might at least understand what was in their hearts. In these circumstances it was felt that there was but one course to pursue. Late on Sunday evening, with much anxious deliberation, a letter was written to Her Royal Highness the Princess of Wales, expressing in a few simple words the tenor of what the nurses had said. At the same time another letter was written to a member of the Royal household setting forth their anxiety to offer some token of their affectionate sympathy wtih the living, and of their pathetic regard for the dead. Many of the nurses, not knowing what else to think of at such a moment, had suggested a wreath or a floral cross. This was mentioned to the Princess on Monday morning as being their wish. Almost immediately a telegraphic message was despatched from Sandringham to London, saying that the floral tribute would be "gratefully accepted," and that it might be sent to Sandringham before the departure of the Prince to his last home.

All nurses will feel that the Princess of Wales, even in the midst of the almost inconsolable grief of a first great bereavement, has had a heart to think of others, and to accept from them, with a gratitude as womanly as it is Royal, the offering of their spontaneous sympathy. If anything could make them think more of their Royal friend and helper than they have hitherto done, it would be some such gracious act as this at such a time.

It will please nurses to know, and not nurses only, that the form their tribute took was that of an Ionic Cross of Violets and Lilies of the Valley, to be placed on the Duke's coffin, accompanied by this simple inscription: "From the Princess of Wales's Nurses of the Royal National Pension Fund, in token of their loving devotion and heartfelt sympathy." Such an offering was but one among many; but it had a value peculiarly its own. It was not the offering of the rich, or of those in exalted station. But it had in it many elements which could not belong to some of the tributes offered by the rich and the great. Private letters were written to about forty hospitals in London and the country, giving nurses the opportunity of offering small subscriptions towards the purchase of the beautiful cross which was to be sent to Sandringham in their names; and within twenty-four hours of the sending of those letters, more than double the amount required had been received. The names of the hospitals whose nurses thus expressed their loyal devotion will be found in another page. Those nurses who yielded to their irresistible longing to offer some token of their deep sympathy and respect to Her Royal Highness, the mother of the dead Prince, will be profoundly touched by the response which their offering called forth at Sandringham. Early on Wednesday morning the following telegram was sent for publication :—

"Will you convey heartfelt thanks to nurses for beautiful and kind tribute to my beloved son's memory." Princess of Wales.

From a member of the Royal household we learn that "the nurses' cross has been all day near the dead Prince in Sandringham Church;" and was to be taken to Windsor with him on the day of the funeral. All the nurses will be grateful, with an unspeakable gratitude, that they have been able to do something which has carried balm to a wounded heart.

Readers, nurses as well as others, would hold that we had failed in our duty if we did not express on their behalf a respectful sympathy for His Royal Highness the Prince of Wales, who has just laid his firstborn in the grave. All the world looks to the mother, because she has not a man's strength wherewith to stand firmly under the stroke of fate. But the father has also a deep and tender heart, even though it be protected by outer walls of masculine tenacity and reason. Those walls are broken through in a moment by the sudden and unlooked-for death of a son in the flower of his youth. The Prince of Wales is a father mourning for his first-born; and let it be remembered that that first-born had never pierced his father's heart by a single undutiful act. Every man who is a father, and every man who has stood by the death-bed of his own first-born, knows what is in the heart of the Prince of Wales to-day, and what will continue to make his heart tender and gentle through all his future years.

The women of our country look with peculiar tenderness towards the Princess May. In six weeks she was to have been married to the husband of her choice; to a young man who, in spite of all temptations to a life of worldly pleasure, had preserved an unsullied heart, and that best of all guarantees of a husband's loyalty, a beautiful and growing affection for his mother. No woman ever had a fairer prospect of happiness than this young woman. If all be true that report utters, no woman ever deserved to have a fairer. In a moment all her fair prospects are dashed to the ground, and no power can ever gather them together again. But for her, too, there is surely consolation. She, with the Prince and Princess of Wales, cannot but feel that the outpoured spirit of a whole nation has power to console. He who was to have been her husband is dead; but he has left a memory which affection may fondly dwell upon, and which will surely become more sacred and tender as the years go by. All men are now beginning to value the dead at his true and modest worth. He was a gentle prince. His grandfather, too, was a gentle prince; but he lived to show that gentleness and greatness may well go hand in hand.

To Her Majesty the Queen the nurses of this country, who owe to her a debt of gratitude and devotion which they can never pay, desire to offer their loyal and respectful sympathy. The Queen stands more and more alone as age advances. She has a mother's love for all her children, to the second and third generation. She was happy in the happiness of the Duke and the Princess May. They were both in a very real sense her children. Once more, in her age, as before in her less ripe years, the stroke of destiny has fallen upon her. May the God of nations console her and be her strength! This is the prayer of all her people, who though dismayed by the sense of their own loss, yet feel that their grief is swallowed up in the Queen's grief, and in that of the father, the mother, and all the Royal House. With them all a nation mourns, and a nation's loyal love would fain bring them consolation.

